# Antifungal Susceptibility Profiles of 1698 Yeast Reference Strains Revealing Potential Emerging Human Pathogens

**DOI:** 10.1371/journal.pone.0032278

**Published:** 2012-03-01

**Authors:** Marie Desnos-Ollivier, Vincent Robert, Dorothée Raoux-Barbot, Marizeth Groenewald, Françoise Dromer

**Affiliations:** 1 Institut Pasteur, Unité de Mycologie Moléculaire, Centre National de Référence Mycologie et Antifongiques, Paris, France; 2 CNRS URA3012, Paris, France; 3 CBS-KNAW Fungal Biodiversity Centre, Utrecht, The Netherlands; Imperial College Faculty of Medicine, United Kingdom

## Abstract

New molecular identification techniques and the increased number of patients with various immune defects or underlying conditions lead to the emergence and/or the description of novel species of human and animal fungal opportunistic pathogens. Antifungal susceptibility provides important information for ecological, epidemiological and therapeutic issues. The aim of this study was to assess the potential risk of the various species based on their antifungal drug resistance, keeping in mind the methodological limitations. Antifungal susceptibility profiles to the five classes of antifungal drugs (polyens, azoles, echinocandins, allylamines and antimetabolites) were determined for 1698 yeast reference strains belonging to 992 species (634 Ascomycetes and 358 Basidiomycetes). Interestingly, geometric mean minimum inhibitory concentrations (MICs) of all antifungal drugs tested were significantly higher for Basidiomycetes compared to Ascomycetes (*p*<0.001). Twenty four strains belonging to 23 species of which 19 were Basidiomycetes seem to be intrinsically “resistant” to all drugs. Comparison of the antifungal susceptibility profiles of the 4240 clinical isolates and the 315 reference strains belonging to 53 shared species showed similar results. Even in the absence of demonstrated *in vitro/in vivo* correlation, knowing the *in vitro* susceptibility to systemic antifungal agents and the putative intrinsic resistance of yeast species present in the environment is important because they could become opportunistic pathogens.

## Introduction

Among the estimated 1.5 million fungal species, approximately 200 species of yeasts and moulds associated with humans as commensals or pathogens are reported, according to the Colloquium of the American Academy of Microbiology on the fungal Kingdom (The Fungal Kingdom: Diverse and Essential Roles in Earth's Ecosystem, June 2008, ASM http://academy.asm.org/). The number of yeasts species has increased over time. In 1928, Guillermond described only 22 genera of yeasts, and the successive editions of one textbook on yeasts included 349 species belonging to 39 genera in 1970 [1], more than 700 species belonging to 100 different genera in 1998 [2], and 11 new genera since 2003 [3], [4]. In the fifth edition of the reference yeast book published by Kurtzman et al. [5], 149 genera and nearly 1500 species are described. Furthermore, new yeast species are regularly recognized as opportunistic pathogens. This can be explained by an increased awareness of invasive fungal infections and by improved techniques that allow better identification of fungal species based on polygenic analyses. There is also an increasing number of patients with various immune defects (haematological malignancies, solid tumours, organ transplantation, immunosuppressive treatments, etc.) or underlying conditions (intensive care unit, indwelling catheter, prosthetic devices, broad spectrum antibiotics, etc.). This complex setting often prompts use of pre-emptive, prophylactic and prolonged curative antifungal treatments that can favour the emergence of resistant species or mutations in the drug target [6]. The azoles are to our knowledge the only class compounds used both in agriculture and in clinical medicine. They block the biosynthesis of fungal ergosterol by inhibition of cytochrome P450 14-alpha demethylase. Derivatives of piperazines, pyridines and pyrimidines are also used as fungicides for plant protection and have a mode of action similar to that of azoles [7].

Knowledge about the yeasts antifungal susceptibility profiles is limited to species responsible for human infections. During this study a comprehensive analysis of antifungal susceptibility profiles of a large number of yeast type strains available at the reference collection of the Centraalbureau voor Schimmelcultures (CBS, Utrecht, The Netherlands) were undertaken. Human pathogenic species as well as species recovered from other sources (*e.g.* environment, food and insects) were included in this study. Antifungal drugs tested belonged to the five known classes of antifungal drugs (polyenes, azoles, echinocandins, allylamines and antimetabolites). The aim of this study was to look into the possibility whether species with decreased susceptibility to one or several classes of antifungal drugs could be a threat for human or animal health taking also into account their ability to grow at 37°C. We also used our database of 4240 isolates recovered mainly from clinical specimens and collected at the French National Reference Center (NRCMA) to compare the antifungal susceptibility of the 53 species shared among the CBS reference strains and the clinical isolates.

## Results

### Differences in antifungal susceptibility profiles between Basidiomycetes and Ascomycetes in the CBS and the National Reference Center for Mycoses and Antifungals (NRCMA) collections

A total of 1698 yeast reference strains and 4240 clinical isolates were studied (Table 1). Table 2 summarizes the antifungal susceptibility results according to the division for all the reference strains and the clinical isolates. For the reference strains, geometric mean minimal inhibitory concentrations (MICs) of all antifungal drugs tested were significantly higher for Basidiomycetes compared to Ascomycetes (*p*<0.001, Table 2). Thus, using the previously defined thresholds, the percentage of reference strains with resistance or decreased susceptibility to drugs was significantly higher for Basidiomycetes than for Ascomycetes (Table 3). Almost half the Basidiomycetes strains could be considered as “resistant” for flucytosine (5FC) and amphotericin B (AmB) and even more than half for fluconazole (FCZ), voriconazole (VRZ), and caspofungin (Caspo). For the Ascomycetes, less than 20% of the reference strains were considered resistant except for FCZ (38.3%). There was no visible alteration in antifungal susceptibility profiles over time for either Basidiomycetes or Ascomycetes (data not shown). If we arbitrarily define “resistance” for a given class of antifungal drug by ≥50% of the strains with decreased susceptibility to that drug, provided that at least five strains were tested, 16 of the 38 genera of reference strains of Ascomycetes tested were “resistant” to at least one class (13 being resistant to azoles), five genera to two classes (two to azoles/5FC, and one each to azoles/echinocandin, azoles/polyenes and azoles/allylamines), and one genus, *Yarrowia*, that has already been described to be a human opportunistic pathogen, to four classes (Table S1). For the Basidiomycetes, all strains representatives of the 19 genera were “resistant” to echinocandins, none was resistant to allylamine; *Fellomyces* and *Hannaella*, that are unknown as human pathogens, were “resistant” to four classes and the strains tested belonging to the 11 genera including the known opportunistic pathogens *Trichosporon*, *Cryptococcus*, *Pseudozyma* and *Rhodosporidium* were resistant to three classes (Table S2).

**Table 1:** Description of the reference strains and the clinical isolates studied.

	Reference strains			Clinical isolates		
	Ascomycetes	Basidiomycetes	Total	Ascomycetes	Basidiomycetes	Total
**Number of**						
**strains**	1209	489	1698	3557	683	4240
**genera**	71	48	119	19	5	23
**No. of strains per genus**	1–385	1–90				
**No. of species per genus**	1–257	1–70				
**species**	634	358	993	43	10	53
**No. of strains per species**	1–30	1–29				
**Site of isolation or substrate**						
**Human**	125	52	177	3489	679	4168
**Living organisms**	503	246	749	3	3	6
**Water**	24	29	53			
**Environment**	185	101	286	6	0	6
**Food & industrial products**	292	19	311			
**unknown**	80	42	122	59	1	60
**Geograpical area** *	994	419	1413			
**Europe**	354	124	478	3493	630	4123
**Africa**	103	18	121	8	6	14
**North America**	171	27	198			
**South America**	138	26	164	55	47	102
**Asia**	195	177	372			
**Oceania**	27	21	48	1	0	1
**Antartica**	6	26	32			
**Period of isolation** *	1193	481	1674			
**1905–1958**	490	109	599			
**1959–1972**	262	84	346			
**1973–1990**	184	101	285			
**1991–1995**	24	24	48			
**1996–2000**	89	41	130			
**2001–2009**	144	122	266	3557	683	4240

* when information is available.

**Table 2:** Distribution of MICs according to the phylum of clinical isolates and reference strains.

	Ascomycetes				Basidiomycetes			
	No. isolates	GeometricMean	MIC50/MIC90 (µg/mL)	Range (µg/mL)	No. isolates	GeometricMean	MIC50/MIC90 (µg/mL)	Range (µg/mL)
**Type strains**								
**Fluconazole**	1147	3.38	4/32	0.12–128	445	14.24	16/128	0.12–128
**Voriconazole**	1147	0.06	0.06/0.5	0.015–16	445	0.24	0.25/4	0.015–16
**Itraconazole**	1147	0.08	0.06/0.05	0.015–16	445	0.20	0.12/16	0.015–16
**Posaconazole**	1147	0.10	0.12/0.5	0.015–16	445	0.22	0.25/2	0.015–16
**Flucytosine**	1147	0.26	0.12/2	0.12–128	445	4.82	4/128	0.12–128
**Terbinafine**	1147	0.84	1/8	0.015–16	445	1.30	2/8	0.015–16
**Caspofungin**	1202	0.09	0.06/0.5	0.015–16	484	4.36	8/16	0.015–16
**Amphotericin B**	1202	0.06	0.06/0.25	0.015–16	484	0.15	0.12/0.5	0.015–16
**Clinical isolates**								
**Fluconazole**	3550	0.98	0.5/32	0.12–128	683	3.90	4/16	0.12–128
**Voriconazole**	3550	0.04	0.015/0.5	0.015–16	683	0.05	0.06/0.12	0.015–16
**Itraconazole**	2206	0.07	0.03/1	0.015–16	427	0.06	0.06/0.25	0.015–4
**Posaconazole**	3547	0.07	0.06/1	0.015–16	683	0.10	0.12/0.25	0.015–16
**Flucytosine**	3550	0.24	0.12/1	0.12–128	683	4.17	4/16	0.12–128
**Terbinafine**	2157	1.27	2/8	0.015–16	420	0.73	1/2	0.03–16
**Caspofungin**	2998	0.06	0.06/0.25	0.015–8	548	3.9	8/8	0.12–8
**Amphotericin B**	3556	0.08	0.06/0.25	0.015–4	682	0.18	0.12/0.5	0.015–16

**Table 3:** Proportion of reference strains with high MIC.

N° of isolates with high MIC* to the corresponding antifungal (%)	Ascomycetes	Basidiomycetes	*p*
**Fluconazole ≥8 µg/mL**	439/1147 (38.3)	302/445 (67.9)	<0.001
**Voriconazole ≥0.25 µg/mL**	236/1147 (20.6)	231/445 (51.9)	<0.001
**Itraconazole ≥0.5 µg/mL**	161/1147 (14.0)	144/445 (32.4)	<0.001
**Posaconazole ≥0.5 µg/mL**	155/1147 (13.5)	149/445 (33.5)	<0.001
**Flucytosine ≥8 µg/mL**	78/1147 (6.8)	219/445 (49.2)	<0.001
**Terbinafine ≥8 µg/mL**	143/1147 (12.5)	80/445 (18.0)	0.006
**Caspofungin ≥0.5 µg/mL**	154/1202 (12.8)	445/484 (91.9)	<0.001
**Amphotericin B ≥0.25 µg/mL**	168/1202 (14.0)	214/484 (44.2)	<0.001
**Amphotericin B ≥1 µg/mL**	25/1202 (2.1)	44/484 (9.1)	<0.001

* Minimal inhibitory concentration.

For the clinical isolates, significant differences in geometric mean MICs were recorded for all drugs between Basidiomycetes and Ascomycetes except for itraconazole (ITZ, not significantly different) and terbinafine (Terbi, MICs were higher in Ascomycetes compared to Basidiomycetes, *p*<0.001) (Table 2).

### Comparison of antifungal susceptibility profiles for the species shared between the collections of CBS reference strains and NRCMA clinical isolates

The MICs for the 53 different species corresponding to the 4240 clinical isolates collected at the NRCMA were analysed and compared to those obtained for the corresponding 315 reference strains (Table 4, Tables S1 and S2). When analysing data for each species (Tables S3 and S4), the overall distribution of the MICs (MIC50, MIC90 and MIC ranges) was similar between reference strains and clinical isolates.

**Table 4:** Comparison of reference strains and clinical isolates with high MIC.

	Ascomycetes					Basidiomycetes				
No. isolates with MIC* to the corresponding antifungal	type strains	%	clinical isolates	%	*p*	type strains	%	clinical isolates	%	*p*
**Fluconazole ≥8 µg/mL**	87/258	33.7	848/3550	23.9	0.001	41/55	74.6	236/683	34.6	<0.001
**Voriconazole ≥0.25 µg/mL**	48/258	18.6	693/3550	19.5	0.745	41/55	74.6	48/683	7.0	<0.001
**Itraconazole ≥0.5 µg/mL**	42/258	16.3	447/2206	20.3	<0.001	37/55	76.3	22/427	5.2	<0.001
**Posaconazole ≥0.5 µg/mL**	46/258	17.8	624/3547	17.6	0.933	34/55	61.8	59/683	8.6	<0.001
**Flucytosine ≥8 µg/mL**	15/258	5.8	184/3550	5.2	0.775	11/55	20	249/683	36.5	0.013
**Terbinafine ≥8 µg/mL**	42/258	16.3	313/2157	14.5	0.457	24/55	43.6	10/420	2.4	<0.001
**Caspofungin ≥0.5 µg/mL**	18/259	7	277/2898	9.6	0.182	53/56	94.6	546/548	99.6	0.007
**Amphotericin B ≥0.25 µg/mL**	53/259	20.5	399/3556	11.2	<0.001	20/56	35.7	337/682	49.4	0.052

* Minimum Inhibitory Concentration.

Among the Ascomycetes, the proportion of decreased susceptibility to antifungals was similar between the reference strains and the clinical isolates for VRZ, posaconazole (PSZ), 5FC, Terbi, and Caspo. A significant but less than 2-fold difference was observed for FCZ and ITZ. The proportion of AmB resistance for reference strains was almost 2-fold more than that recorded for clinical isolates (Table 4). One *Yarrowia lipolytica* reference strain and two clinical isolates of *Candida glabrata* and *Candida krusei*, were resistant to all five classes of antifungal drugs. Major discrepancies were observed for *Candida tropicalis* with MIC50 and MIC90 of the azoles higher for the reference strains than for the clinical isolates where seven from the12 reference strains were resistant to at least one azole, including six recovered before 1970 and four from human samples. None of the *C. tropicalis* reference strains had 5FC MIC>8 µg/ml in contrast with almost 33% (103/313) of the clinical isolates. Some discrepancies in Caspo MICs distribution were also noted with a few clinical isolates of *Candida albicans*, *Candida glabrata*, *Candida rugosa*, *Candida tropicalis*, *Clavispora lusitaniae* and *Kodamaea ohmeri* that exhibited high MICs. Most of these isolates have been recovered from patients previously exposed to echinocandins.

Among the Basidiomycetes the percentage of strains with resistance or decreased susceptibility was drastically higher among reference strains than among clinical isolates for azoles and Terbi, and lower for 5FC and AmB. Of the 604 Basidiomycetes tested (548 clinical isolates and 56 reference strains), only two *Cryptococcus neoformans*, one *Cryptococcus gattii*, one *Cryptococcus laurentii* and one *Trichosporon inkin* had low Caspo MICs, leaving 99% of the Basidiomycetes resistant to Caspo.

### Relationship between thermotolerance, source of isolation and antifungal drugs susceptibility profile

Among the 1470 reference strains for which the maximum temperature of growth was determined, 535 (36.4%) were known to grow at 37°C or above, including 488 Ascomycetes (48 genera and 227 species) and 47 Basidiomycetes (8 genera and 26 species). Therefore, the proportion of strains able to grow at 37°C varied according to the Division (46.3% for the Ascomycetes and 11.3% for the Basidiomycetes) and also according to their source of isolation (from 61.6% for strains of human source to 16.7% for those from soil, Table 5) with no Basidiomycetes that were recovered from insects were able to grow at 37°C. Among the 535 thermotolerant strains, 271 (50.7%; *i.e.* 256 Ascomycetes strains belonging to 177 species in 45 genera; 15 Basidiomycetes strains belonging to 15 species in eight genera) belonged to species unknown as human pathogens, or at least not described in articles available in the NCBI library (http://www.ncbi.nlm.nih.gov/). The proportion of strains with decreased susceptibility to the various antifungal drugs varied according to the source of isolation (humans, other living organisms, food and industrial products, environment and water): azoles (39±14%), 5FC (11±8%), Terbi (13±4%), Caspo (23±12%) and AmB (20±8%) (Table 5). Strains recovered from insects had the lowest proportion of “resistance” compared to other sources for all antifungals except 5FC (data not shown). The highest proportion of “resistance” was observed for strains recovered from drinks (alcoholic beverages, fruit juices, soft drinks) (with regards to azoles and Terbi resistance), from humans (5FC), environment and animals (Caspo) and animals (AmB).

**Table 5:** Reference strains able to grow at 37°C with high MIC according to source of isolation.

		Percent of strains with decreased susceptibility to									
Source of isolation	No. strains able to grow at 37°C/No. strains	No. Strains Tested in RPMI	Fluco	Vori	Itra	Posa	5FC	Terbi	No. Strains Tested in AM3	Caspo	AmB
**Human**	109/177	109	28%	20%	12%	10%	16%	11%	109	21%	17%
**Living organisms**	188/749	183	32%	20%	13%	11%	9%	9%	188	15%	17%
**Environment**	58/286	56	45%	21%	18%	16%	5%	16%	58	17%	17%
**Water**	9/53	9	0%	11%	11%	0%	22%	11%	9	44%	33%
**Food & Industrial products**	140/311	130	50%	25%	23%	25%	4%	19%	140	16%	19%
**All (mean ± SD)**	504/1556	487	31±20	19±5	15±5	12±9	11±8	13±4	504	23±12	20±8

When analysing all the reference strains regardless of the thermotolerance, 24 strains (*i.e.* 1.5% of the 1580 strains tested against all antifungal drugs), belonging to 23 different species and including 19 Basidiomycetes, were resistant to all drugs. The reported sources were six strains from the environment, five from soil, six from plants and one from animal and human. Among the 23 species, two were recognized human opportunistic pathogens (*Cryptococcus albidus* and *Y. lipolytica*), and three were recovered from clinical specimens (*Cryptococcus magnus*, *Bulleromyces albus* (CBS database) and *Pseudozyma hubeiensis* (NRCMA unpublished data)). Among the four Ascomycetes isolates resistant to all antifungal drugs, three were included or related to the *Y. lipolytica* clade (*Y. lipolytica*, *Candida hispaniensis* and *Candida umkomasiana*). Among these 24 “resistant” strains, 17 were able to grow at 30°C and only seven at 37°C. Finally, among the seven resistant species able to grow at 37°C, five were unknown as human pathogen (two Ascomycetes, *C. hispaniensis* and *Zygosaccharomyces mellis*; three Basidiomycetes, *Pseudozyma crassa*, *Trichosporon lactis* and *Trichosporon veenhuisii*).

## Discussion

The limitations of the present study should be kept in mind. Firstly, antifungal susceptibility testing was not performed for all strains in identical conditions because of changes in incubation parameters requested by temperature requirements and slow growth rate in the test media for some species. This implies also that not all reference strains of a given genus and/or only a few strains for a given species were available for analysis. Secondly, storage of the reference strains was uneven considering time period, geographical zone and source of isolation, thus preventing definitive conclusions regarding evolution of drug resistance over time or according to substrates. Furthermore, the proportion of Ascomycetes versus Basidiomycetes from the various substrates for the reference strains does not reflect the reality in terms of overall species' diversity, colonization, saprophitism or pathogenicity. Finally, future changes in taxonomy may alter some of our conclusions that are based on our current knowledge on specific genera or species.

The first striking result of this study was the higher proportion of strains with decreased susceptibility, regardless of the class of antifungal drugs used, among Basidiomycetes compared to Ascomycetes reference strains. The drastic difference observed for echinocandins (92% Basidiomycetes and 13% Ascomycetes reference strains with Caspo MIC≥0.5 µg/ml) is well known for pathogenic species, and was confirmed by comparison with the clinical isolates. Caspofungin is one of the three antifungal agents in the class of echinocandins. The target of the echinocandins is the synthetic cell-wall enzyme complex beta-1,3D-glucansynthase [8]. *Filobasidiella neoformans* had a hotspot region in beta-1,3D-glucansynthase similar to that of the susceptible Ascomycetes species *C. albicans* and *Saccharomyces cerevisiae* but is intrinsically resistant to echinocandins. A previous study has already shown the presence of *fks1*-like genes in ten species of Basidiomycetes with sequences remarkably similar to Fks1 from *F. neoformans* [9]. This difference in echinocandins susceptibility between Ascomycetes and Basidiomycetes could be the result of phylogenetic differentiation of the cell-wall composition with potential modification of the three-dimensional structure of the betaglucan synthase complex that remains to be analyzed.

Almost all (84%) of the genera of Basidiomycetes included more than 50% of reference strains with decreased susceptibility to azoles compared to 34% of the genera of Ascomycetes. Most (68%) genera of Basidiomycetes included more than 50% of the reference strains with decreased susceptibility to at least three classes compared to the Ascomycetes genera (3%). The fact that the resistance profiles of the reference strains were not associated with a specific substrate or period corresponding to specific drug launching time suggest that it may not have been acquired through environmental pressure but rather through recombination or other evolutionary processes. A comprehensive analysis of the target genes is necessary to validate this hypothesis.

The comparison of the antifungal susceptibility profiles of the reference strains with the clinical isolates belonging to shared species showed that the profiles were similar except for a few species. Indeed, some *Candida* spp. clinical isolates with high Caspo MIC were recovered from patients previously exposed to Caspo (acquisition of resistance by nonsense mutation in hotspot regions of the betaglucan synthase [10], [11], [12], [13]), whereas reference strains of *C. albicans*, *C. glabrata*, *C. rugosa*, *C. tropicalis*, *C. lusitaniae* and *K. ohmeri* recovered before Caspo usage had low MICs. None of the *C. tropicalis* type strains had 5FC MIC>8 µg/ml in contrast with almost 33% of the clinical isolates. It was previously showed that most of these clinical isolates belong to a single 5FC-resistant clone [14]. The widespread use of antifungal drugs especially azoles since the 1970s in agriculture (http://www.pesticides.gov.uk/rags.asp?id=644) may also contribute to the emergence of resistance. The drugs are used repeatedly over a long period of time and could thereby create a persistent pressure of azole compounds on saprophytic fungi [15]. Indeed, resistance of clinical isolates of *Aspergillus* spp. to azoles has been shown to occur in patients treated with voriconazole but also following environmental exposure of the infecting isolates [13], [16]. However, there is no obvious explanation for the high proportion of *C. tropicalis* reference strains with high azoles MICs compared to very few clinical isolates since only three were recovered after 1970. However, the overall similarity of the results between reference strains and clinical isolates, suggests that the results obtained with the other reference strains could be valid.

Robert and Casadevall suggested in their study of fungal thermotolerance that “new human pathogenic fungi are likely to emerge from genera that are already tolerant to higher temperatures” [17]. Among the 1698 reference strains studied, 535 strains from 253 species were able to grow at human body temperature, among which several species (177/634 Ascomycetes species and 15/358 Basidiomycetes species) were unknown as human opportunistic pathogens. Keeping in mind that growth limitation evidenced *in vitro* could be related to culture medium or conditions and could not apply *in vivo*, it was found that five of the seven species were resistant to all antifungal classes and able to grow at 37°C were unknown as human pathogen. Even though the *in vitro/in vivo* correlation was not demonstrated, knowing the *in vitro* susceptibility to systemic antifungal agents and the putative intrinsic resistance of these thermotolerant species is important as they could become opportunistic human pathogens. As an example, the species *Y. lipolytica* has been studied for its biotechnological importance [18] but is also known to be responsible for human invasive infections mainly in immunocompromised patients. Interestingly, among the six species belonging to the *Y. lipolytica* clade [19], the two species *Y. lipolytica* and *C. hispaniensis*, both able to grow at 37°C, include two reference strains with high MICs for all antifungals. *Candida hispaniensis*, described in 2005, is still unknown as human or animal opportunist. Furthermore, among the *Y. lipolytica* clade, *Candida bentonensis*, able to grow at 35°C, had high MICs for all antifungals except 5FC. *Candida galli*, previously described in chicken infections, and unable to grow above 35°C, had high MICs for 5FC, Fluco, Vori and AmB. The other species of the clade, *Candida uncommunis* and *Aciculoconidium aculeatum* were unable to grow above 35°C and 30°C respectively and had high MICs for AmB. Of note, the isolate of the species *C. umkomasiana*, previously identified as *Y. lipolytica* was also resistant to all antifungal drugs. The genomic study of species present in the *Y. lipolytica* clade and of additional closely related species could therefore be particularly interesting in terms of multidrug resistance.

In conclusion, this study provides the first comprehensive analysis of antifungal susceptibility profiles for almost 1700 reference strains representing more than 119 genera and 993 species. It paves the way to further phylogenetic studies on the origin of drug resistance and evolution in Basidiomycetes and Ascomycetes. It also shows that species with high levels of resistance can be found in the environment without previous environment drug pressure and could become opportunistic human pathogens. Finally, it underlines how important correct identification of species is, especially for uncommon species, pointing out the need for reliable fungal sequence databases [20].

## Materials and Methods

### Yeasts Type Strains

A total of 1698 yeast reference strains available from the CBS collection, that include type strains (T), authentic strains (AUT), syntypes (ST), lectotypes (LT), IT (isotype), AT (allotype) or neotypes (NT) of currently accepted species and also from previously described species that are now considered to be synonyms of the accepted species, were studied. The strains belonged to the Division of Ascomycota (71.2%, 71 genera, 634 species) and Basidiomycota (28.8%, 48 genera, 358 species) (Table 1). Strains were recovered from various sources, such as plants (27.7%), soil (12.4%), drinks (10.7%), insects (10.6%) and humans (10.4%) (Table 1). The geographical areas of isolation were diverse, but most strains were isolated from Europe (33.8%) and Asia (26.3%) for the 1413 isolates for which the information was available. The oldest isolate was found in 1905, but 43.5% of the strains were recovered since 1973 and 15.9% since 2001.

All strains were stored in lyophilised form at the CBS. They were subcultured at least twice on Sabouraud agar plates (Gibco®, Invitrogen™, Life Technologies, CA, USA) before *in vitro* susceptibility testing.

### Clinical isolates

The NRCMA received clinical isolates from short-stay university hospital laboratories. These isolates are part of surveillance programs or send to the NRCMA due to difficulties during the identification process. The isolates corresponding to species for which a reference strain was available were included in the analysis (n = 4240) (Table 1). They belonged to the Ascomycota (83.9%, 19 genera and 43 species) and Basidiomycota (16.1%, five genera, ten species). Five species encompass 86.3% of the clinical isolates (*Candida albicans* (40.9%), *Cryptococcus neoformans* (14.9%), *Candida glabrata* (14.5%), *Candida parapsilosis* (8.6%) and *Candida tropicalis* (7.4%)). Isolates were mainly recovered from humans (83.7%) in Europe (84.7%) and all between January 2003 and July 2010.

Upon receipt, all isolates were identified by phenotypic methods (urease activity, Urea-Indole, bioMérieux, Marcy l'Etoile, France; carbon assimilation profiles, ID32C, bioMérieux), and by sequencing of the ITS and D1/D2 regions of the gene coding for ribosomal RNA by using universal primers (V9D/LS266 and NL1/NL4 primers, respectively [21], [22], [23], [24]). *Candida albicans* was differentiated from *Candida dubliniensis* by amplification of a portion of the gene coding for actin [25]. MICs of various systemic antifungal drugs (see below) were determined on a routine basis for each isolate.

### *In vitro* susceptibility testing

*In vitro* susceptibility was determined by a microdilution technique following the procedure proposed by the Antifungal Susceptibility Testing Subcommittee of EUCAST (AFST-EUCAST [26]) with modifications of the temperatures and duration of incubation when necessary.

Active powder of known potency of flucytosine (5FC, Sigma, Saint Quentin Fallavier, France), fluconazole (FCZ, Pfizer Inc., Groton, CT, USA), itraconazole (ITZ, Janssen-Cilag, Issy-les-Moulineaux, France), voriconazole (VRZ, Pfizer), posaconazole (PSZ, Merck and Co., Rahway, New Jersey, USA), terbinafin (Terbi, Novartis Pharma AF, Basel, Schwitzerland), caspofungin (Caspo, Merck) and amphotericin B (AmB, Sigma Chemicals) were used. Stock solutions were prepared in sterile distilled water (5FC, FCZ, Caspo, Terbi) or DMSO (AmB, ITZ, VRZ, PSZ) and stored at −80°C until used. Stock solutions were diluted in RPMI 1640 (Sigma) buffered to pH 7.0 with 0.165 M morpholinepropanesulfonic acid (MOPS, Sigma) for all antifungals except Caspo and AmB that were diluted in AM3 medium (Difco™, Becton-Dickinson, Le Pont de Claix, France). Final concentrations ranged from 0.125 to 64 µg/ml (5FC and FCZ) and from 0.015 to 8 µg/ml (for the others). Both media were supplemented with glucose (final concentration 2%). Microplates (96 wells flat-bottom, TPP®, Switzerland) were prepared by batches and stored frozen at −20°C for less than 6 months. The following reference strains were included as quality controls: *C. parapsilosis* ATCC 22019 and *Pichia kudriavzevii* (synonym of *Issatchenkia orientalis*, teleomorph of *Candida krusei* [27]) ATCC6258. Fungal growth was determined by an automated microplate reader spectrophotometer (Multiscan RC-351, Labsystems Oy, Helsinki, Finland) at 492 nm after at least 24 h of incubation and/or until absorbance of the drug-free well was ≥0.2. The MIC was defined as a 50% (90% for amphotericin B) or more reduction in growth compared to the drug-free well.

For the reference strains, a few parameters had to be changed because the procedure is standardized for glucose fermentative yeasts able to grow at 35°C. Thus, growth temperature for the subculture and subsequent incubation of the microplates was changed for the majority of the strains (30°C, 20°C and 15°C for 1503, 158, and 17 strains, respectively). Incubation time varied, 24 hrs for 713 (42%), 48 hrs for 466 (27%), 72 hrs for 232 (14%), 4 days for 145 (8.5%), 6 days for 121 (7%), 7 days for 21 (1%) strains. Likewise, the test medium did not allow sufficient growth and thus determination of the MIC for some strains was limited to azoles, 5FC and Terbi or Caspo and AmB (106 missing values for drugs tested in RPMI and 12 for drugs tested in AM3).

Resistance to FCZ and VRZ were defined as a MIC higher than or equal to 8 and 0.25 µg/mL, respectively according to EUCAST recommendations [26]. Decreased susceptibility to 5FC and Caspo were arbitrarily defined by a MIC higher than or equal to 8 and 0.5 µg/mL, respectively according to previous data obtained in the laboratory showing that clinical isolates of *Candida* spp. exhibiting MICs above these thresholds harboured mutations in target genes [28], [29]. We defined arbitrary thresholds for ITZ, PSZ, AmB and Terbi by determining the MIC that inhibited at least 90% of the clinical isolates. This corresponded to a MIC higher than or equal to 0.5 µg/mL for ITZ and PSZ, ≥0.25 µg/mL for AmB and ≥8 µg/mL for Terbi. The values corresponding to the concentration inhibiting 50% (MIC50) or 90% (MIC90) of the strains were calculated for species including more than ten strains.

### Statistical analysis

Data were analyzed using Stata™ (version 10.1, College Station, TX, USA). Distribution of MICs between Ascomycetes and Basidiomycetes were compared using the non-parametric Kruskall Wallis test. Comparisons between groups were done using Chi Square or Fisher exact tests for the proportions of strains/isolates with decreased susceptibility to a drug. A *p*-value of <0.05 was considered statistically significant.

## Supporting Information

Table S1 Percentage of isolates with high MIC within genera in the Ascomycota phylum.
(XLS)

Table S2 Percentage of “resistant” isolates within genera in the Division Basidiomycota.
(XLS)

Table S3 Comparison of the distribution of MICs between clinical isolates and reference strains from the 53 shared species for azoles.
(XLS)

Table S4 Comparison of the distribution of MICs between clinical isolates and reference strains from the 53 shared species for Amphotericine B, Flucytosine, Terbinafine and Caspofungine.
(XLS)
